# Favorable prognosis of breast cancer brain metastases patients with limited intracranial and extracranial metastatic lesions

**DOI:** 10.1186/s13014-023-02293-6

**Published:** 2023-07-01

**Authors:** Wei Shi, Yang Li, Hua Sun, Li Zhang, Jin Meng, Xiaofang Wang, Xingxing Chen, Xiaomeng Zhang, Xin Mei, Jinli Ma, Miao Mo, Changming Zhou, Fei Liang, Zhimin Shao, Zhen Zhang, Xiaomao Guo, Xiaoli Yu, Zhaozhi Yang

**Affiliations:** 1 Department of Radiation Oncology, Fudan University Shanghai Cancer Center, 270 Dong-An Road, Shanghai, 200032 China; 2 Department of Oncology, Shanghai Medical College, Fudan University, Shanghai, 200032 China; 3 Shanghai Clinical Research Center for Radiation Oncology, Shanghai Key Laboratory of Radiation Oncology, Shanghai, 200032 China; 4 Affiliated Hospital of Jining Medical University, Jining, 272029 Shandong China; 5 Department of Cancer Prevention and Clinical Statistics Center, Fudan University Shanghai Cancer Center, Shanghai, 200032 China; 6 Department of Biostatistics, Zhongshan Hospital, Fudan University, Shanghai, China; 7 Department of Breast Surgery, Fudan University Shanghai Cancer Center, Shanghai, 200032 China

**Keywords:** Brain metastases, Breast cancer, Oligometastases, Systemic therapy, Salvage local therapy

## Abstract

**Background:**

Breast cancer brain metastases (BCBM) are highly heterogenous with widely differing survival. The prognosis of the oligometastatic breast cancer (BC) patients with brain metastases (BM) has not been well studied. We aimed to investigate the prognosis of BCBM patients with limited intracranial and extracranial metastatic lesions.

**Methods:**

Four hundred and forty-five BCBM patients treated between 1st January 2008 and 31st December 2018 at our institute were included. Clinical characteristics and treatment information were obtained from patient’s medical records. The updated breast Graded Prognostic Assessment (Breast GPA) was calculated.

**Results:**

The median OS after diagnosis of BM were 15.9 months. Median OS for patients with GPA 0–1.0, 1.5–2, 2.5–3 and 3.5–4 were 6.9, 14.2, 21.8, 42.6 months respectively. The total number of intracranial and extracranial metastatic lesions, in addition to the Breast GPA, salvage local therapy and systemic therapy (anti-HER2 therapy, chemotherapy and endocrine therapy) were demonstrated to be associated with prognosis. One hundred and thirteen patients (25.4%) had 1–5 total metastatic lesions at BM diagnosis. Patients with 1–5 total metastatic lesions had a significantly longer median OS of 24.3 months compared to those with greater than 5 total metastatic lesions with a median OS of 12.2 months (*P *< 0.001; multivariate HR 0.55, 95% CI, 0.43–0.72). Among the patients with 1–5 metastatic lesions, median OS for GPA 0–1.0 was 9.8 months, compared to 22.8, 28.8 and 71.0 for GPA 1.5–2.0, 2.5–3.0 and 3.5–4.0 respectively, which is much longer than the corresponding patients with greater than 5 total metastatic lesions, with medium OS of 6.8, 11.6, 18.6 and 42.6 months respectively for GPA 0–1.0, 1.5–2.0, 2.5–3.0 and 3.5–4.0.

**Conclusions:**

The patients with 1–5 total metastatic lesions demonstrated better OS. The prognostic value of the Breast GPA and the survival benefit of salvage local therapy and continuation of systemic therapy after BM were confirmed.

**Supplementary Information:**

The online version contains supplementary material available at 10.1186/s13014-023-02293-6.

## Introduction

Breast cancer (BC) is the second most common cause of brain metastases (BM) [1]. Among patients diagnosed with BC, approximately 15% eventually develop BM and the incidence is likely to grow with advances in systemic therapy, more routine surveillance imaging and improvements in imaging techniques [2]. Human epidermal growth factor receptor 2 (HER2)-positive and triple-negative metastatic BC have highest incidence of BM, with the incidence rate of up to 50% [3–5].

Over decades, treatment has changed dramatically and survival has improved significantly [6]. Local therapies for BM include surgery, whole-brain radiotherapy (WBRT) and stereotactic radiosurgery (SRS). The choice of local treatments depends on factors such as prognosis of patients, presence of symptoms, number and size of BM, resectability, prior therapy and extent of metastases. Advances in systemic therapy, especially anti-HER2 therapy, resulted in improved intracranial response rates and prolonged OS. If the effective systemic therapy is used, local therapies may be delayed until there is intracranial progression [7–11].

With advances in treatment, it is acknowledged that breast cancer brain metastases (BCBM) are highly heterogenous with widely differing survival instead of having uniformly dismal prognosis. Potential prognostic factors include performance status, age, tumor subtype, number of BM, the presence of extracranial metastases and receipt of local or systemic therapy. The updated breast Graded Prognostic Assessment (Breast GPA) is one of the most well-known prognostic indices, among various other prognostic indices [12, 13]. The Breast GPA consisted of five prognostic factors: KPS, subtype, age, number of BM, the presence of extracranial metastases [6].

Accumulating evidence supported the existence of an oligometastatic state which was characterized by limited total tumor burden and restricted tumor metastatic capacity [14, 15]. Metastasis-directed treatment (MDT) could potentially cure oligometastatic patients. Most of the current randomized trials for oligometastatic BC [16–18] excluded patients with BM. The prognosis of the oligometastatic BC patients with BM has not been well studied and the prognostic value of total metastatic lesions was not fully explored.

To the best of our knowledge, so far no studies exist focusing on the prognostic value of total number of both intracranial and extracranial metastases in BCBM patients. Therefore, we aimed to investigate the prognosis of patients with 1–5 versus greater than 5 total metastatic lesions in a large single center cohort.

## Materials and methods

### Patient selection and data collection

Four hundred and forty-five female patients were included in the study, among biopsy-proven BC patients who were radiologically diagnosed as BM between 1 January 2008 and 31 December 2018 at our institute. Data were censored as of June 30, 2021. Patients with unconfirmed BM, concurrent leptomeningeal metastases and incomplete information were excluded (Additional file 1: Figure S1). Patient information including age, histology, subtype, Karnofsky Performance Score (KPS), number of BM, extracranial metastases, treatment received and other patient characteristics was extracted from patient’s medical records. The total number of metastases were recorded based on imaging data retrospectively. The study protocol was reviewed and approved by the institutional ethics committee of our institute. Written informed consent was obtained from all patients.

### Statistical analysis

The overall survival (OS) after BM was defined as the time from initial BM diagnosis to the date of death or last follow-up. Data were censored as of June 30, 2021. The Kaplan–Meier method was used to estimate OS. The Breast GPA scores for each patient was calculated and the patients were divided into four bands (0–1.0, 1.5–2.0, 2.5–3.0 and 3.5–4.0) [6]. Univariate (UVA) and multivariate analysis (MVA) using the Cox proportional hazard model were performed to investigate the prognostic factors of OS. In multivariate analyses, forward conditional strategy was used for building the model. The potential prognostic factors analyzed include: total number of both intracranial and extracranial metastases, year of diagnosis of BM, time from diagnosis of BC to diagnosis of recurrence or BM, KPS, hormone receptor (HR) and HER2 status, age at diagnosis of BM, number of BM, extracranial disease, symptoms of BM and local/systemic therapy after diagnosis of BM. All clinicopathological features and treatment modalities tested in the univariate analysis were included in the multivariate analyses. The Breast GPA, instead of its five components (KPS, subtype, age, number of BM, the presence of extracranial metastases), was included as one factor in the multivariate analyses. A significant difference was considered when *P* < 0.05. All statistics were calculated using statistical package for the social sciences (SPSS®) 26.0 software (SPSS Inc., Chicago, IL, USA). The Kaplan–Meier survival curves were plotted by using the R package.

## Results

### Clinical characteristics

Four hundred and forty-five female patients were included in the study. Among them, 249 (56.0%) were diagnosed in the 1st time period (2008–2014) and the rest 196 (44.0%) were diagnosed in the 2nd period (2015–2018). The Breast GPA scores differentiated patients into four groups. Overall, 103 patients (23.1%) had a Breast GPA score of 0–1.0, 182 patients (40.9%) had a GPA score of 1.5–2.0, 141 (31.7%) patients had a GPA score of 2.5–3.0 and 19 (4.3%) had a GPA score of 3.5–4.0. At BM diagnosis, KPS of ≤ 60 was found in 21.3% (n = 95) patients, 70–80 was found in 70.6% (n = 314) patients and 90–100 was found in 8.1% (n = 36). The most common histological type at diagnosis of primary breast cancer is ductal carcinoma (94.9%, n = 371). The most common molecular subtype was HR + /HER2- (30.1%, n = 128), followed by HR-/HER2-(24.9%, n = 106), HR + /HER2 + (23.8%, n = 101), and HR-/HER2 + (21.2%, n = 90). The median age was 52 years at first diagnosis of BM (range, 28–81 years) and median relapse time between diagnoses of BC and BM was 35 months (range, 0–273 months). At BM diagnosis, single brain lesion developed in 32.4% patients (n = 144) and extracranial disease was found in the majority of patients (83.4%, n = 371). The most common extracranial metastatic sites included lung (n = 222 lesions), bone (n = 205 lesions), lymph nodes (n = 200 lesions) and liver (n = 130 lesions). At BM diagnosis, 35.0% (n = 154) patients had no clinical symptoms. At BM diagnosis, 198 patients (44.5%) had equal to or less than 3 organs involved. Approximately one quarter of patients (25.4%, n = 113) had equal to or less than 5 total metastatic lesions including brain metastases.

The vast majority of patients (98.1%, n = 424) received local therapy after BM diagnosis. The most common local treatment was WBRT alone (60.4%, n = 261), followed by WBRT + three-dimensional conformation radiotherapy (3DCRT)/SRS (19.9%, n = 86) and SRS alone (10.9%, n = 47) (Table 1). Over time, more patients received SRS and less patients received WBRT. The patients who received SRS increased from 4.4% (n = 11) to 18.4% (n = 36), from the 1st time period (2008–2014) to the 2nd period (2015–2018), whereas the patients who received WBRT decreased from 69.9% (n = 174) to 44.4% (n = 87) correspondingly. Salvage local therapy for locally recurrent brain metastases was delivered to 48 (10.8%) patients, which included SRS (n = 35), WBRT (n = 10) and surgery (n = 3). Patients who received salvage local therapy increased from 7.2% (n = 18) to 15.3% (n = 30) from the 1st time period (2008–2014) to the 2nd period (2015–2018).

**Table 1:** Characteristics of BCBM patients (n = 445)

Parameter	Category	Overall
Treatment era	2008–2014	249 (56.0%)
	2015–2018	196 (44.0%)
Breast GPA score	0.0–1.0	103 (23.1%)
	1.5–2.0	182 (40.9%)
	2.5–3.0	141 (31.7%)
	3.5–4.0	19 (4.3%)
Histological type (primary breast cancer)	Ductal	371 (94.9%)
	Lobular	8 (2.0%)
	Other	12 (3.1%)
	Missing	54
Time from diagnosis of breast cancer to recurrence	Median (range)	22 (0–256)
	≤ 24 months	248 (55.7%)
	> 24 months	197 (44.3%)
Time from diagnosis of breast cancer to brain metastasis	Median (range)	35 (0–273)
	≤ 24 months	136 (30.6%)
	> 24 months	309 (69.4%)
KPS at diagnosis of brain metastasis	≤ 60	95 (21.3%)
	70–80	314 (70.6%)
	90–100	36 (8.1%)
Tumor subtype (primary breast cancer)	HR-/HER2-	106 (24.9%)
	HR+/HER2-	128 (30.1%)
	HR−/HER2+	90 (21.2%)
	HR+/HER2+	101 (23.8%)
	Missing	20
Age at diagnosis of brain metastasis (years)	Median (range)	52 (28–81)
	≥ 60	94 (21.1%)
	< 60	351 (78.9%)
Number of brain metastasis	Multiple lesions	301 (67.6%)
	Single lesion	144 (32.4%)
Extracranial disease	Yes	371 (83.4%)
	No	74 (16.6%)
Extracranial metastatic sites (not mutually exclusive)	Liver	130
	Lung	222
	Bone	205
	Lymph nodes	200
	Breast or chest wall	41
	Serous membrane effusion	46
	Adrenal gland	13
	Other^a^	8
Number of involved organs	≤ 3	198 (44.5%)
	> 3	247 (55.5%)
Number of metastatic lesions	≤ 5	113 (25.4%)
	> 5	332 (74.6%)
Asymptomatic brain metastases	Yes	154 (35.0%)
	No	286 (65.0%)
	Missing	5
Local therapy after brain metastasis	Surgery ± WBRT/SRS	30 (6.9%)
	SRS alone	47 (10.9%)
	WBRT alone	261 (60.4%)
	WBRT + 3DCRT/SRS	86 (19.9%)
	No local therapy	8 (1.9%)
	Missing	13
Salvage local therapies	No	397 (89.2%)
	Yes	48 (10.8%)
Chemotherapy	With Capecitabine	184 (45.0%)
	Without Capecitabine	144 (35.2%)
	No chemotherapy	81 (19.8%)
	Missing	36
Anti-HER2 therapy	Trastuzumab ± Pertuzumab	48 (10.8%)
	TKI(Pyritinib/Lapatinib) ± Trastuzumab	97 (21.9%)
	Other	3 (0.7%)
	HER2 + without anti-HER2 therapy	40 (9.0%)
	HER2- without anti-HER2 therapy	255 (57.6%)
	Missing	2
Endocrine therapy	Tamoxifen	6 (1.3%)
	AI ± OFS	43 (9.7%)
	AI + CDK4/6 inhibitor ± OFS	9 (2.0%)
	Other	13 (2.9%)
	HR + without endocrine therapy	209 (47.0%)
	HR- without endocrine therapy	165 (37.1)

^a^Other includes kidney (n= 3 lesions), skin (n = 2 lesions), spleen (n = 2 lesions) and pancreas (n =1 lesion)

*WBRT* whole-brain radiotherapy, *SRS* stereotactic radiosurgery, *3DCRT* three-dimensional conformation radiotherapy, *HR* hormone receptor (estrogen and/or progesterone receptors, *HER2* human epidermal receptor 2, *AI* aromatase inhibitor, *OFS* ovarian function suppression, *CDK* cyclin-dependent kinase

There have been major achievements in the treatment of BC during the study period ranging from 2008 to 2018, such as the introduction of tyrosine kinase inhibitor (TKI) and Pertuzumab. Most patients received chemotherapy (80.2%, n = 328), 78.7% HER2 positive patients (148 out of 188) received anti-HER2 therapy and 25.4% HR positive patients (71 out of 280) received endocrine therapy after diagnosis of BM (Table 1). The number of Her2 positive patients who did not receive anti-HER2 therapy decrease from 30.7% (27 out of 88) in the 1st time period (2008–2014) to 13.0% (13 out of 100) in the 2nd period (2015–2018). The HER2 positive patients who received TKI (Pyritinib/Lapatinib) ± Trastuzumab after diagnosis of BM increased from 35.6% (n = 32) to 65.0% (n = 65) from the 1st time period (2008–2014) to the 2nd period (2015–2018).

### Survival analysis

**Fig. 1:**
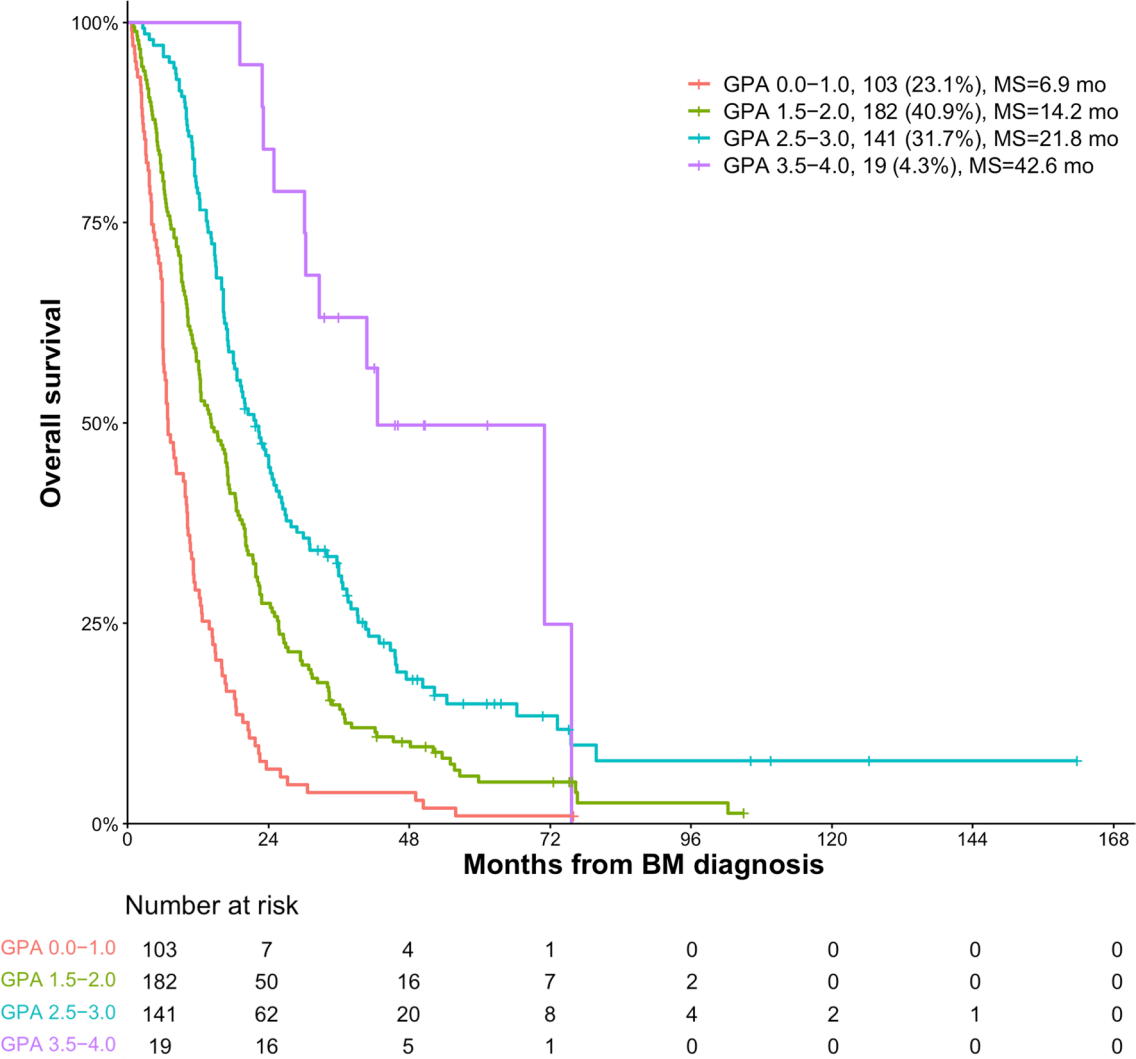
Kaplan–Meier curve stratified by GPA band. MS: median survival. mo: months

At the time the data were recorded in June 30, 2021, 41 of the 445 patients were still alive. The median OS after BM was 15.9 months (95% confidence interval [CI], 14.3–17.5 months), with 6 months, 1 year, 2 years, 3 years, 4 years, 5 years OS rates of 81.6%, 59.6%, 30.5%, 19.4%, 12.9% and 8.6% respectively. OS after BM was compared in two time periods, 2008–2014 and 2015–2018. In the UVA, the OS during the 2nd period (2015–2018) was significantly longer than the 1st period (2008–2014); The medium OS was 14.3 months (95% CI, 11.8–16.8) during the 1st time period compared with 16.8 months (95% CI, 13.4–20.2) during the 2nd time period (*P* < 0.05) (Table 2). The Kaplan–Meier curve for survival using GPA scores demonstrated an excellent separation between GPA bands (*P* < 0.001). Median OS for patients with GPA 0–1.0 was 6.9 months, compared to 14.2, 21.8 and 42.6 for GPA 1.5–2.0, 2.5–3.0 and 3.5–4.0 respectively (Table 2 and Fig. 1). The Breast GPA, the total number of both intracranial and extracranial metastases, salvage local therapy for locally recurrent brain metastases and continued systemic treatment after BM (anti-HER2 therapy, chemotherapy and endocrine therapy) were demonstrated to be significantly correlated with OS in both the UVA and MVA analysis (*P* < 0.05; Tables 2 and 3).

**Table 2:** Univariate cox proportional hazards model for overall survival

Variable	Category	Median OS (95% CI)	Hazard ratio (95% CI)	*P* value
Year of diagnosis of brain metastasis	2008–2014	14.3 (11.8–16.8)	1	
	2015–2018	16.8 (13.4–20.2)	0.74 (0.61–0.91)	0.003
Breast GPA score	0.0–1.0	6.9 (5.3–8.5)	1	< 0.001
	1.5–2.0	14.2 (11.2–17.2)	0.49 (0.38–0.64)	< 0.001
	2.5–3.0	21.8 (17.7–25.9)	0.30 (0.23–0.39)	< 0.001
	3.5–4.0	42.6 (20.1–65.1)	0.14 (0.07–0.26)	< 0.001
KPS at diagnosis of brain metastasis	≤ 60	6.9 (5.6–8.2)	1	< 0.001
	70–80	16.7 (15.1–18.3)	0.45 (0.35–0.57)	< 0.001
	90–10	39.3 (28.2–50.4)	0.19 (0.12–0.30)	< 0.001
Tumor subtype (primary breast cancer)	HR-/HER2-	10.2 (7.8–12.6)	1	< 0.001
	HR+/HER2-	13.7 (9.8–17.6)	0.81 (0.62–1.05)	0.115
	HR−/HER2+	16.3 (13.4–19.2)	0.65 (0.49–0.87)	0.004
	HR + /HER2+	22.7(18.2–27.2)	0.48 (0.36–0.65)	< 0.001
	Missing	22.5 (0–50.1)	0.52 (0.31–0.85)	0.01
Age at diagnosis of brain metastasis	≥ 60	14.8 (9.7–19.9)	1	
	< 60	15.9 (14.2–17.6)	0.94 (0.75–1.19)	0.628
Number of brain metastasis	Multiple lesions	13.1 (11.4–14.9)	1	
	Single lesion	19.2 (15.2–23.2)	0.60 (0.48–0.75)	< 0.001
Extracranial disease	Yes	14.5 (12.6–16.4)	1	
	No	22.8 (19.4–26.2)	0.58 (0.44–0.77)	< 0.001
Interval between breast cancer to recurrence	≤ 24 months	14.9 (12.6–17.2)	1	
	> 24 months	16.6 (13.7–19.5)	0.94 (0.77–1.15)	0.54
Interval between breast cancer to brain metastasis	≤ 24 months	14.8 (11.6–18.0)	1	
	> 24 months	16.3 (14.4–18.2)	0.98 (0.79–1.21)	0.816
Number of involved organs	≤ 3	20.0 (17.2–22.8)	1	
	> 3	11.4 (9.5–13.3)	1.61 (1.32–1.97)	< 0.001
Number of metastatic lesions	> 5	12.2 (10.7–13.7)	1	
	≤ 5	24.3 (20.9–27.7)	0.46 (0.37–0.59)	< 0.001
Asymptomatic brain metastasis	No	14.2 (12.1–16.3)	1	0.228
	Yes	18.4 (16.2–20.5)	0.86 (0.38–0.63)	0.141
	Missing	13.8 (7.6–20.0)	1.22 (0.51–3.00)	0.655
Salvage local therapies	No	14.1 (12.1–16.1)	1	
	Yes	25.8 (22.3–29.3)	0.51 (0.36–0.71)	< 0.001
Chemotherapy	No	5.3 (4.3–6.3)	1	< 0.001
	Yes	18.6 (16.8–20.4)	0.35 (0.27–0.45)	< 0.001
	Missing	10.6 (8.4–12.8)	0.66 (0.44–0.98)	< 0.001
Anti-HER2 therapy	HER2+ without anti-HER2 therapy	10.2 (6.8–13.6)	1	< 0.001
	HER2- without anti-HER2 therapy	12.2 (10.0–14.4)	1.01 (0.72–1.42)	0.952
	Trastuzumab ± Pertuzumab	18.4 (11.7–25.1)	0.63 (0.40–0.98)	0.039
	TKI(Pyritinib/Lapatinib) ± Trastuzumab	21.8 (17.6–26.0)	0.58 (0.39–0.86)	0.006
	Other	17.2 (11.4–23.0)	0.91 (0.28–2.94)	0.871
	Missing	12.3 (N/A)	0.99 (0.24–4.11)	0.99
Endocrine therapy	HR + with endocrine therapy	25.0 (20.9–29.1)	1	< 0.001
	HR + without endocrine therapy	12.4 (10.2–14.6)	1.84 (1.38–2.47)	0.001
	HR negative	15.9 (13.4–18.4)	1.70 (1.25–2.29)	< 0.001

*HR* hormone receptor (estrogen and/or progesterone receptors); *HER2* human epidermal receptor 2

**Table 3:** Multivariable cox proportional hazards model for overall survival

Variable	HR	95% CI	*P* value
*GPA*			
0.0–1.0	1		< 0.001
1.5–2.0	0.63	0.48–0.84	0.002
2.5–3.0	0.35	0.23–0.53	< 0.001
3.5–4.0	0.17	0.08–0.36	< 0.001
*Number of metastatic lesions*			
> 5	1		
≤ 5	0.55	0.43–0.72	< 0.001
Salvage local therapy			
No	1		
Yes	0.58	0.41–0.83	0.003
*Chemotherapy*			
No	1		< 0.001
Yes	0.24	0.17–0.33	< 0.001
Missing	0.40	0.25–0.64	< 0.001
*Anti-HER2 therapy*			
HER2+ with anti-HER2 therapy	1		0.030
Her2+ without anti-HER2 therapy	1.49	1.01–2.20	0.047
HER2−	0.84	0.61–1.16	0.297
*Endocrine therapy*			
HR+ with endocrine therapy	1		< 0.001
HR+ without endocrine therapy	2.88	2.03–4.06	< 0.001
HR negative	2.87	2.02–4.09	< 0.001

*HR* hormone receptor (estrogen and/or progesterone receptors); *HER2* human epidermal receptor 2

### The effect of metastatic lesions on OS

**Fig. 2:**
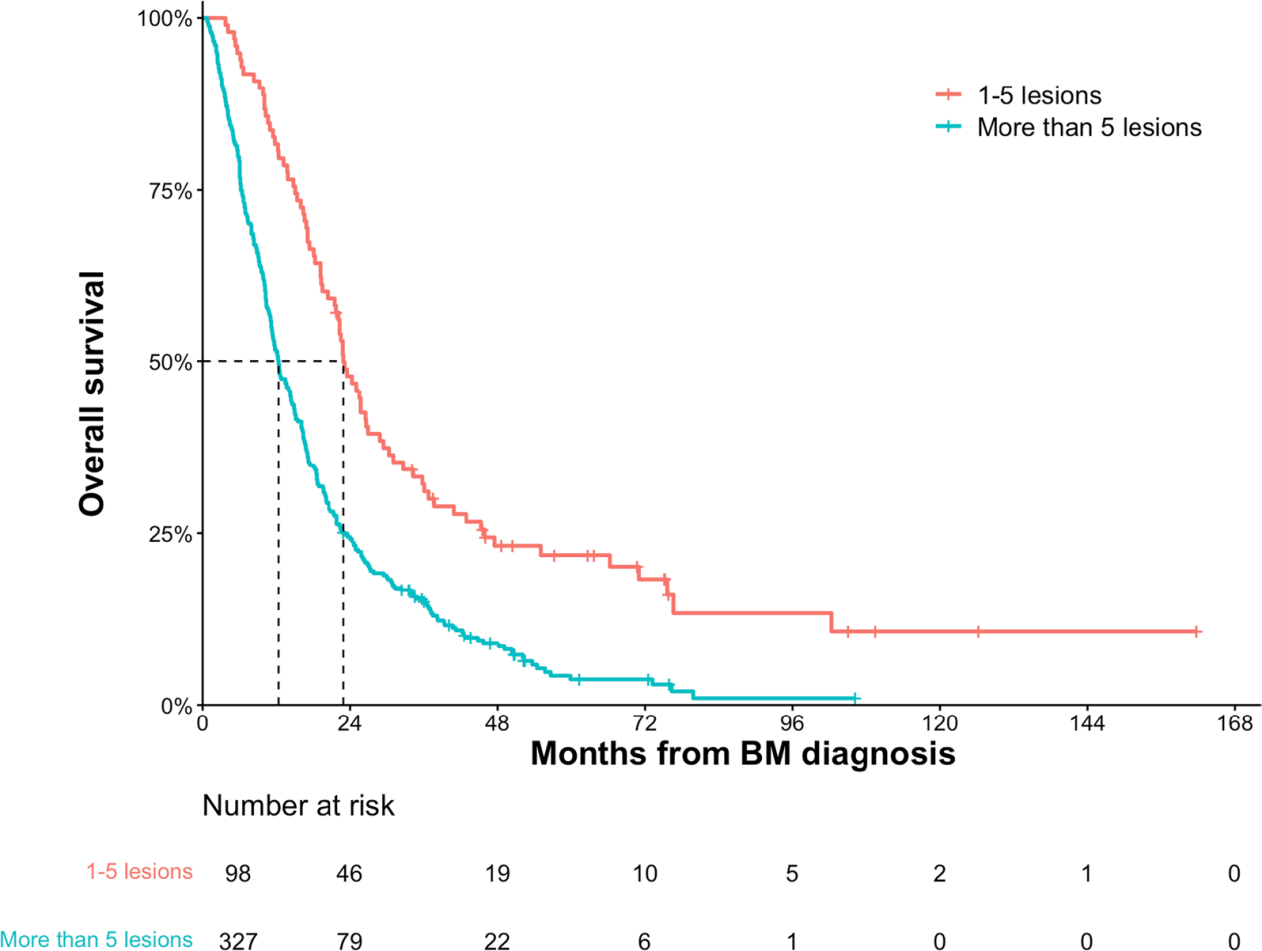
Overall survival of patients with 1–5 metastatic lesions versus more than 5 metastatic lesions

**Fig. 3:**
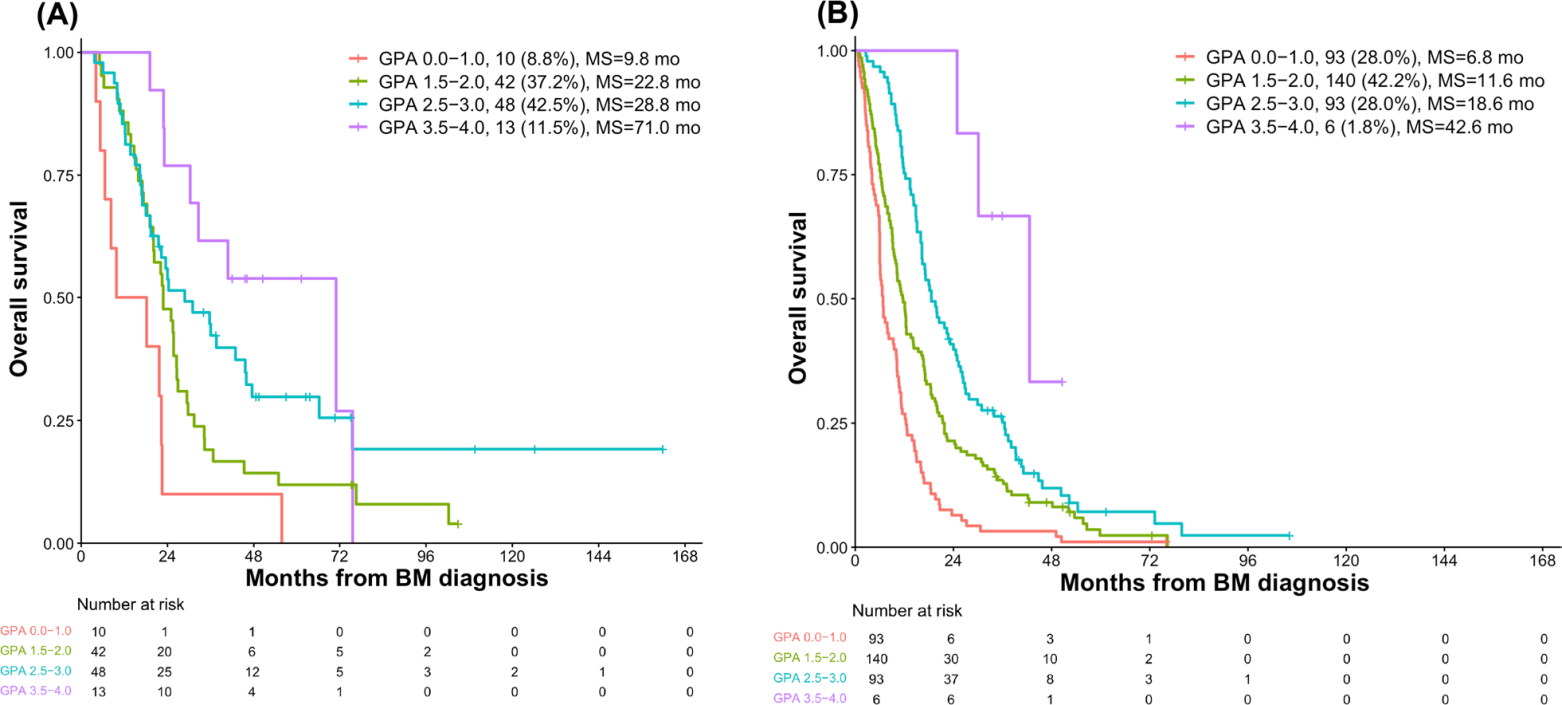
Kaplan–Meier curve stratified by GPA band in patients with 1–5 total metastatic lesions (**A**) and with greater than 5 total metastatic lesions (**B**). MS: median survival. mo: months

The total number of both intracranial and extracranial metastatic lesions (> 5 versus ≤ 5) at diagnosis of BM was shown to be a significant predictor of OS in both the UVA and MVA (*P* < 0.001; multivariate hazard ratio [HR] 0.55, 95% CI, 0.43–0.72; Tables 2 and 3). Patients with 1–5 metastatic lesions had a significantly longer median OS of 24.3 months (95% CI, 20.9–27.7) compared to those with greater than 5 total metastatic lesions who had a median OS of 12.2 months (95% CI, 10.7–13.7) (Table 2 and Fig. 2). At 5 years, the estimated OS was 22.3% (95% CI, 18.2–26.4) in the group of 1–5 metastatic lesions and 3.7% (95% CI, 2.5–4.9) in the group of greater than 5 total metastatic lesions, respectively (Fig. 3). Among the patients with 1–5 metastatic lesions (n = 113), median OS for patients with GPA 0–1.0 (n = 10, 8.8%) was 9.8 months, compared to 22.8, 28.8 and 71.0 for GPA 1.5–2.0 (n = 42, 37.2%), 2.5–3.0 (n = 48, 28.8%) and 3.5–4.0 (n = 13, 11.5%) respectively (Fig. 3A), which is much longer than the corresponding patients with greater than 5 total metastatic lesions, with medium OS of 6.8, 11.6, 18.6 and 42.6 months respectively for GPA 0–1.0 (n = 93, 28.0%), 1.5–2.0 (n = 140, 42.2%), 2.5–3.0 (n = 93, 28.0%) and 3.5–4.0 (n = 6, 1.8%) (Fig. 3B).

Among the patients with 1–5 total metastatic lesions (n = 113), 50.4% (n = 57) patients had extracranial metastases. For the extracranial metastases, only 3.5% (n = 4) patients received radical local therapy (surgery of lung or liver metastases for 1 patient each, fractioned stereotactic radiation therapy [FSRT] of liver or bone metastases for 1 patient each).

### Salvage local therapy and continued systemic therapy

In addition to the Breast GPA and the total number of metastatic lesions, salvage local therapy and continued systemic therapy (anti-HER2 therapy, chemotherapy and endocrine therapy) were demonstrated to be associated with prognosis.

**Fig. 4:**
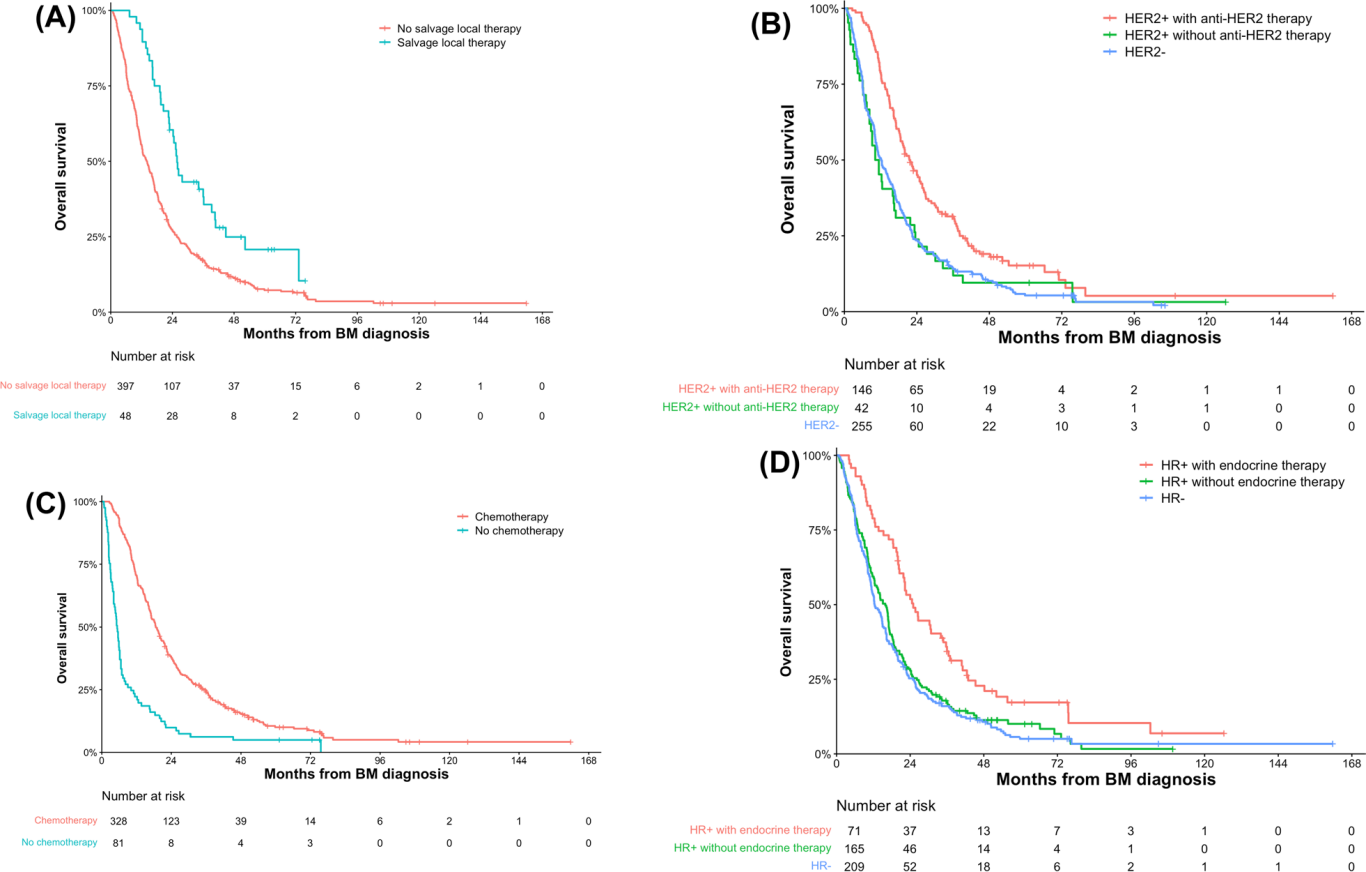
Kaplan–Meier curves for overall survival stratified by salvage local therapy (**A**), anti-HER2 therapy (**B**), chemotherapy (**C**) and endocrine therapy (**D**). HR: hormone receptor (estrogen and/or progesterone receptors); HER2: human epidermal receptor 2

Salvage local therapy for locally recurrent brain metastases resulted in better OS. Patients who received salvage local therapy had a significantly longer medium OS of 25.8 months (95% CI, 22.3–29.3) compared to those without salvage local therapy who had a medium OS of 14.1 months (95% CI, 12.1–16.1), both in the UVA and MVA (*P* < 0.05, multivariate HR 0.58, 95% CI, 0.41–0.83) (Tables 2, 3 and Fig. 4A).

In addition, our study indicated that continued systemic treatment (anti-HER2 therapy, chemotherapy and endocrine therapy) after BM resulted in an improved OS compared with no systemic therapy. HER2 positive patients who were treated with Trastuzumab ± Pertuzumab or TKI(Pyritinib/Lapatinib) ± Trastuzumab had a significantly longer medium OS (18.4 and 21.8 months, respectively) than those without anti-HER2 therapy (10.2 months) in UVA (*P* < 0.05). In MVA, HER2 positive patients who received anti-HER2 therapy had a significantly longer medium OS than those without anti-HER2 therapy (*P* < 0.05, multivariate HR 1.49, 95% CI, 1.01–2.20; Table 3 and Fig. 4B). Besides, patients who received chemotherapy after BM had a significant longer medium OS of 18.6 months (95% CI, 16.8–20.4) than those without chemotherapy who had a medium OS of 5.3 months (95% CI, 4.3–6.3) (*P* < 0.001, multivariate HR 0.24, 95% CI, 0.17–0.33; Tables 2, 3 and Fig. 4C). In terms of endocrine therapy, our study showed that endocrine therapy in HR positive patients after BM resulted in an improved OS compared with no endocrine therapy (25.0 months versus 14.1 months) (*P* < 0.001, multivariate HR 2.88, 95% CI, 2.03–4.06; Tables 2, 3 and Fig. 4D).

## Discussion

In this cohort study spanning 10 years, we investigated the survival and prognostic factors of BCBM patients and validated the predictive value of the Breast GPA. Our study demonstrated an increase of 2.5 months in OS during the 2nd time period (2015–2018) compared to the 1st time period (2008–2014). Continued systemic treatment after BM and salvage local therapy after intracranial progression were associated with an improved survival. Besides, the Breast GPA was confirmed to be predictive of OS [6]. The medium OS for patients with GPA 3.5–4.0 was 42.6 months. In addition, the prognosis of oligometastatic BCBM patients was analyzed. BCBM patients with less than or equal to 5 total metastatic lesions had favorable prognosis.

Zhang et al. reported a medium OS of 12 months in a previous study which enrolled 101 BCBM patients treated between 2006 and 2010 with WBRT and systemic therapy at our institute [19]. This study demonstrated an increase in OS over time, with medium OS of 14.3 months (95% CI, 11.8–16.8) during the 1st time period compared with 16.8 months (95% CI, 13.4–20.2) during the 2nd time period. The continued increase in OS is possibly partly due to advancement in local and systemic therapy. Our study indicated that systemic treatment after BM improved OS. The survival benefit of anti-HER2 therapy and chemotherapy was observed, which was consistent with previous retrospective studies [19–25]. In addition, we also found that endocrine therapy after BM diagnosis prolonged the survival of HR positive patients, which was consistent with a previous study [26]. The effect of systemic treatment may be caused by the control of extracranial metastases, increase in permeability of blood–brain barrier (BBB) after WBRT and the use of systemic therapy agents that could penetrate the BBB [9, 27].

Another prognostic factor in this study is salvage local therapies. Salvage local therapies after intracranial progression included neurosurgery, SRS and WBRT. Salvage local therapies were widely employed, utilized in about 44–87% BM patients as reported in previous studies [28–32]. Our study indicated that salvage therapies for locally recurrent brain metastases was correlated with better OS. This could be explained by the fact that some patients only had intracranial progression after the effective systemic treatment. More patients received salvage local therapies in the 2nd period, which may in some degree reflect more active management of locally recurrent BM over time.

Current evidence-based guidelines emphasize the importance of patients’ stratification, in order to optimally individualize the management of BCBM patients [33–35]. The Breast GPA is one of the most well-known prognostic indices and was updated in 2020 with a larger contemporary cohort. The prognostic factors in the updated Breast GPA were KPS, subtype, age, number of BM and the presence of extracranial metastases [6]. Our study demonstrated that median OS for patients with GPA 0–1, 1.5–2, 2.5–3 and 3.5–4 were 6.9, 14.2, 21.8, 42.6 months respectively, consistent with OS reported in the updated Breast GPA (6.0, 12.9, 23.5, 36.3, respectively). Medium OS for patients in the best prognostic group was more than 3 years.

The prognosis of the oligometastatic BC patients with BM has not yet been well studied. In addition, the survival benefit of MDT for oligometastatic BC is inconclusive [16, 36]. Several clinical trials for oligometastatic BC excluded patients with BM [16–18] except for OLIGOMA [37] specifically for BC and SABR-COMET [36, 38, 39] for all cancer types. Our study showed that the BCBM patients with 1–5 total metastatic lesions had a median OS of 24.3 months and 22.3% patients survived more than 5 years. HER2 was overexpressed in 43.4% of these patients. Advances in systemic therapy, especially anti-HER2 therapy, resulted in an improved intracranial response rates and prolonged OS [7–11]. Radiotherapy to the brain may be delayed until there is intracranial progression [7–11]. In this context, the importance of radiotherapy to the brain appeared to be underestimated [40].

To the best of our knowledge, this was the first study that analyzed the prognosis of the oligometastatic BC patients with BM. Oligometastatic BCBM patients have favorable prognosis and should not be excluded from the clinical trials of oligometastatic BC. However, this study had several limitations. First, the study was retrospective in nature with inherent flaws such as selection bias and heterogeneity of the data. The combination, sequence and timing of systemic therapy (anti-HER2 therapy, chemotherapy, endocrine therapy) and local therapy varied widely between patients. Therefore, any conclusions of the efficacy of these interventions warrant cautious interpretation. Second, the oligometastatic disease was defined as 1–5 total metastatic lesions, solely based on imaging findings in this study. In addition to total number of metastatic lesions, other clinical and biological characteristics to define oligometastases should be explored in future studies. Third, only a small proportion of “oligometastatic” BCBM patients received curative local therapy for intracranial and extracranial metastases in this study. The results from clinical trials which treat all metastases with radical local therapy (eg. SBRT, surgery) and most up-to-date systemic therapy, are needed to determine the prognosis of these patients [14, 15].

## Supplementary Information

**Additional file 1. Figure S1:**.Consolidated Standards of Reporting Trialsdiagram illustrating the selection and exclusion of patients

## Data Availability

The data that support the findings of this study are available from the corresponding author, upon reasonable request.

## Abbreviations

BCBM: Breast cancer brain metastases
BM: Brain metastases
BC: Breast cancer
WBRT: Whole-brain radiotherapy
SRS: Stereotactic radiosurgery
Breast GPA: The updated breast Graded Prognostic Assessment
MDT: Metastasis-directed treatment
KPS: Karnofsky Performance Score
OS: Overall survival
UVA: Univariate analysis
MVA: Multivariate analysis
3DCRT: Three-dimensional conformation radiotherapy
TKI: Tyrosine kinase inhibitor
HER2: Human epidermal growth factor receptor 2
HR: Hormone receptor
BBB: Blood–brain barrier
HR: Hazard ratio
CI3: Confidence interval
