# Supplementary material for: Favorable prognosis of breast cancer brain metastases patients with limited intracranial and extracranial metastatic lesions
Source: Radiat Oncol. 2023 Jul 1;18:107. doi: 10.1186/s13014-023-02293-6 (PMC10314440; doi:10.1186/s13014-023-02293-6)
Supplement: Supplementary file 1 — Additional file 1. Figure S1:.Consolidated Standards of Reporting Trialsdiagram illustrating the selection and exclusion of patients [file 13014_2023_2293_MOESM1_ESM.pptx]

## Slide 1
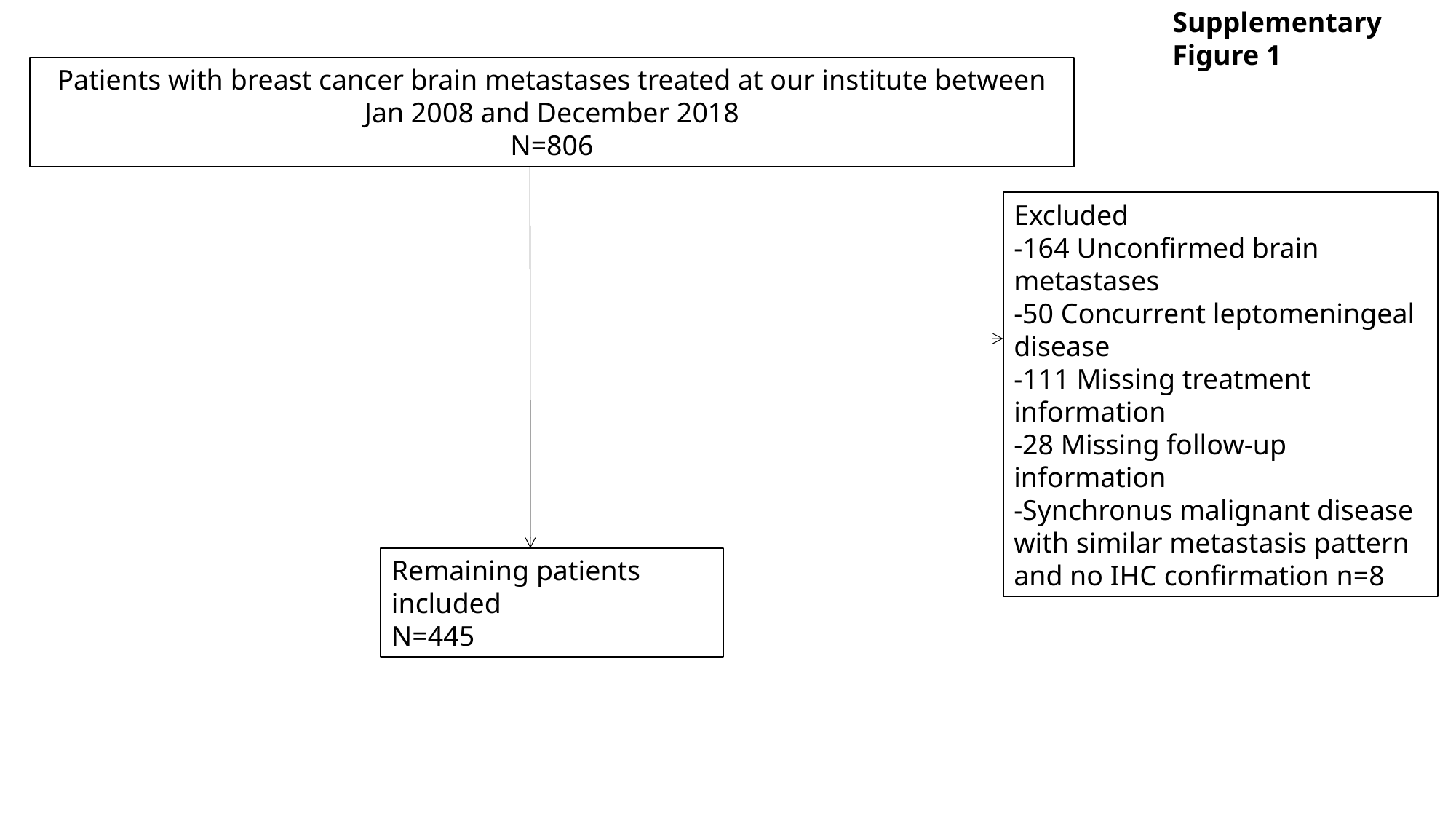

Supplementary Figure 1
Patients with breast cancer brain metastases treated at our institute between Jan 2008 and December 2018
N=806
Excluded
-164 Unconfirmed brain metastases
-50 Concurrent leptomeningeal disease
-111 Missing treatment information
-28 Missing follow-up information
-Synchronus malignant disease with similar metastasis pattern and no IHC confirmation n=8
Remaining patients included
N=445
